# Long-Term Mootral Application Impacts Methane Production and the Microbial Community in the Rumen Simulation Technique System

**DOI:** 10.3389/fmicb.2021.691502

**Published:** 2021-10-08

**Authors:** Johanna Brede, Manuela Peukert, Björn Egert, Gerhard Breves, Melanie Brede

**Affiliations:** ^1^Institute for Physiology and Cell Biology, University of Veterinary Medicine Hannover, Hanover, Germany; ^2^Department of Safety and Quality of Meat, Max Rubner-Institut, Federal Research Institute of Nutrition and Food, Kulmbach, Germany; ^3^Department of Safety and Quality of Fruit and Vegetables, Max Rubner-Institut, Federal Research Institute of Nutrition and Food, Karlsruhe, Germany

**Keywords:** methane, cattle, garlic, microbial community, RUSITEC

## Abstract

Methane emissions by ruminants contribute to global warming and result in a loss of dietary energy for the animals. One possibility of reducing methane emissions is by dietary strategies. In the present trial, we investigated the long-term effects of Mootral, a feed additive consisting of garlic powder (*Allium sativum*) and bitter orange extracts (*Citrus aurantium*), on fermentation parameters and the microbial community in the rumen simulation technique (RUSITEC) system. The experiment lasted 38 days and was divided into three phases: an equilibration period of 7 days, a baseline period (BL) of 3 days, and experimental period (EP) of 28 days. Twelve fermentation vessels were divided into three groups (*n* = 4): control (CON), short-term (ST), and long-term (LT) application. From day 11 to day 27, 1.7 g of Mootral was added to the ST vessels; LT vessels received 1.7 g of Mootral daily for the entire EP. With the onset of Mootral application, methane production was significantly reduced in both groups until day 18. Thereafter, the production rate returned to the initial quantity. Furthermore, the short chain fatty acid fermentation profile was significantly altered by Mootral application; the molar proportion of acetate decreased, while the proportions of propionate and butyrate increased. Metabolomic analysis revealed further changes in metabolite concentrations associated with the Mootral supplementation period. The methyl coenzyme-M reductase gene copy number was reduced in the liquid and solid phase, whereas the treatment did not affect the abundance of bacteria. At the end of the BL, Methanomicrobia was the most abundant archaeal class. Mootral supplementation induced an increase in the relative abundance of *Methanomassiliicoccales* and a reduction in the relative abundance of Methanomicrobia, however, this effect was transient. Abundances of bacterial families were only marginally altered by the treatment. In conclusion, Mootral has the transient ability to reduce methane production significantly due to a selective effect on archaea numbers and archaeal community composition with little effect on the bacterial community.

## Introduction

In the rumen, methanogenic archaea produce methane mainly by reducing CO_2_ with hydrogen (Morgavi et al., 2010). Additionally, formate, methanol, and methylamines serve as substrates for methanogenesis (Hungate et al., 1970; Patterson and Hespell, 1979). Methane production depends on various factors such as carbohydrate intake and composition, rumen retention time, rate of fermentation, and methanogenesis (Beauchemin, 2009). Reducing enteric methane emissions is desirable to reduce the contribution of livestock to greenhouse gas emissions.

Many strategies to reduce methane emissions have been evaluated, which can be differentiated into dietary and non-dietary approaches. The latter are, for example, defaunation (Machmuller et al., 2003), anti-methanogenic vaccination (Wedlock et al., 2010), and breeding of ruminants with low methane emissions (Attwood et al., 2011). Moreover, various dietary strategies have been examined regarding their anti-methanogenic effect. Firstly, there have been attempts to alter the diet in grain content, forage type, and quality (Haque, 2018). Secondly, the supplementation of lipids (Johnson and Johnson, 1995), and secondary plant compounds such as saponins (Lila et al., 2003) and tannins (Tavendale et al., 2005) have been tested. Furthermore, organic acids for example fumaric acid have been investigated (Riede et al., 2013). Moreover, the supplementation of chemicals such as 3-nitrooxypropanol (Martinez-Fernandez et al., 2018) and ionophores (Odongo et al., 2007) have been studied. These strategies either target the methanogens or alter metabolic processes so that less substrate is available for methanogenesis (Haque, 2018). Many of the described approaches have the potential to inhibit methane production, but their effectiveness is limited by practicability, decreased palatability or a negative impact on productivity. According to Beauchemin (2009), the ideal strategy is highly dependent on the particular farm, geographic region, feed, and the type of animal.

Among the dietary approaches, application of garlic and flavonoids has been the focus of many studies. Garlic contains allicin and is known for its antimicrobial properties (Ankri and Mirelman, 1999). The potential of garlic to reduce methane production has been widely studied *in vitro* (Busquet et al., 2005; Soliva et al., 2011; Mbiriri et al., 2017) and *in vivo* (Patra et al., 2011; Ma et al., 2016). Flavonoids from citrus fruits are a group of polyphenols with antioxidative, antimicrobial und anti-inflammatory properties (Kumar and Pandey, 2013). Hence, these plant compounds have also been investigated regarding their effect on methane production (Oskoueian et al., 2013).

Mootral (Mootral SA, Rolle, Switzerland) is a feed additive, which consists of garlic powder (*Allium sativum*) and bitter orange extracts (*Citrus aurantium*). The strong potential of Mootral to mitigate methane production has already been demonstrated *in vitro* by Eger et al. (2018). In this study, a 1 and 2 g/days application of Mootral were evaluated during 1 week of supplementation in the rumen simulation technique (RUSITEC)-system. Both doses substantially reduced the percentage of methane in the fermentation gas (by 95 and 99%, respectively). In a recently published study by Ahmed et al. (2021) application of Mootral at a level of 20% of the substrate led to a reduction of methane proportion up to 54% in a batch culture. In an *in vivo* study by Vrancken et al. (2019) a pellet with 3% Mootral powder reduced methane emissions in Jersey cows by 38% and in Holstein Friesian cows by 21%. In another study by Roque et al. (2019), Angus × Hereford cross steers were supplemented with 15 g of Mootral per day for a duration of 12 weeks. A reduction in methane production by 23% was reported.

However, although the effectiveness of Mootral in reducing methane production *in vitro* and *in vivo* has already been demonstrated, the mechanisms which lead to methane reduction still remain unclear. In the present study, we aimed to investigate the impact of a long-term Mootral application on fermentation parameters, methane production and its effects on the microbial community within the RUSITEC-system. Therefore, we intensively studied both, liquid- and solid-associated archaea and bacteria and identified specific metabolite alterations using an untargeted metabolomics approach.

## Materials and Methods

### Ethics Statement

The fistulation of the donor cows was permitted by the Lower Saxony State Office for Consumer Protection and Food Safety (LAVES, Oldenburg, Germany) under AZ 33.19-42502-05-13A373. In this project, all activities including animals were carried out under the requirements of the German Animal Welfare Act.

### Experimental Setup

In 1977, Czerkawski and Breckenridge introduced the RUSITEC (Czerkawski and Breckenridge, 1977). For this experiment, two systems with six fermentation vessels each were applied, and all fermenters were fed with the same basal diet of 7 g of hay (70.1% of diet dry matter) and 3 g of concentrate (29.9% of dry matter, deuka Schaffutter, Deutsche Tiernahrung Cremer GmbH & Co. KG, Bramsche, Germany, Supplementary Table 1) per day. The same hay and concentrate was fed to the donor cows. The hay was cut into pieces of approximately 1 cm length. The trial lasted 38 days and was designed to compare the short- and long-term effects of Mootral (Mootral SA, Rolle, Switzerland, crude nutrients are presented in Supplementary Table 1). The product was produced as described by Eger et al. (2018). The powder used in this trial contained 1% allicin with a stability of 6 months. The experiment started 4 weeks after delivery of the product. The experiment consisted of three phases: equilibration (days 0–7), baseline period (BL, days 8–10), and experimental period (EP, days 11–38). The equilibration period was performed to ensure a stable microbial community composition in the system, and the BL aimed to verify that there were not differences in fermentation among the treatment groups before supplementation. The 12 vessels were divided into three treatment groups (each *n* = 4): control (CON), short-term (ST), and long-term (LT) application. The CON group remained untreated during the entire experiment. In the LT group, the diet was supplemented with 1.7 g Mootral (inclusion rate: 17.7% of the diet dry matter content) for the entire EP, in the ST group 1.7 g was supplied from the beginning of the EP (day 11) until day 27, by adding Mootral into the feed bags.

At the start of the experiment, rumen fluid, and solid contents of two rumen-fistulated non-lactating German Black Pied cattle were collected as an inoculum 3 h after the morning feeding. The cows were fed a basal diet of 6.5 kg hay, 600 g concentrate, and 100 g minerals (VitaMineral Trockensteher, Agravis, Münster, Germany) once per day. The rumen fluid was removed from each cow’s rumen and filled in a bottle *via* a pump. Solid rumen contents were collected from the solid phase in the dorsal rumen sac. Rumen contents were transported from the stable to the laboratory in closed well-filled containers to avoid extensive exposure to oxygen. Liquid contents from each cow were filtered through a gauze (Lohmann & Rauscher International GmbH & Co. KG, Rengsdorf, Germany) to remove larger feed particles and mixed in another bucket. Afterward, 660 mL of mixed rumen fluid was filled into each of the fermentation vessels. During the whole procedure, rumen contents were kept warm using a water bath (39°C). The solid rumen content was squeezed out through gauze and a total of 70 g was weighed into each of the 12 nylon bags (R712 Forage Bags *in situ*, ANKOM Technology, 6.75 cm × 12 cm, Gesellschaft für Analysetechnik HLS, Salzwedel, Germany). One of these inoculum bags was placed into the inner vessel of each of the fermentation vessel together with another nylon bag containing the formerly described basal diet (feed bag). The whole procedure was finished about 45 min after collection of rumen contents. A motor ensured a permanent up and down movement (6 times/min) of the inner vessel. A modified McDougall’s buffer (Wetzels et al., 2018) was infused into the fermentation vessel using a pump (Typ B1, Ole Dich, Hvidore, Denmark) with a dilution rate of 0.77 L/days. At day 1, the inoculum bag was replaced after 24 h of incubation by a feed bag. At day 2, the feed bag from day 0 was exchanged for a new one. Thereafter, the feed bags were changed alternately, so that each feed bag remained in the fermentation vessel for 48 h. After removing the feed or inoculum bag from the fermentation vessel, it was transferred to a plastic bag and 40 mL of the modified McDougall’s buffer was added. The bag was washed by squeezing for 60 s to detach the microorganisms loosely associated with the feed particles. Subsequently, the washing liquid was added back to the vessels. The daily overflow was collected in a glass flask *via* a butyl tube (8.0 mm × 12.0 mm, YMC Europe GmbH, Dinslaken, Germany). Glass flasks were placed in a styrofoam box filled with ice, to stop further fermentation processes. Daily, nitrogen (Widmann Gase GmbH, Elchingen, Germany) was infused into the overflow flasks after removing the fluid to maintain anaerobic conditions. To seal the overflow flask, a plug with a further butyl tube was placed into the aperture. The butyl tube was linked to a gas bag (Plastigas^®^, Linde AG, Pullach, Germany) to collect the fermentation gas for further evaluation of the gas volume. Gas samples for analysis of the gas composition were collected in a glass bulb (Pfeuffer, Hannover, Germany) installed between flask and gas bag.

### Sampling and Measurements

Measurements of pH (Polyplast pH Sensor, Hamilton Bonaduz AG, CH-7402, Switzerland), redox potential (Polyplast ORP, Hamilton Bonaduz AG, connected with a Digital-pH-Meter 646, Knick GmbH & Co. KG, Berlin, Germany), and overflow volume were carried out daily for the entire duration of the experiment. Overflow samples for determining short chain fatty acid (SCFA) and ammonia-N concentrations were collected daily in BL and every fourth day during EP. The samples were stored at −18°C until further processing. The SCFA concentrations were analyzed by gas chromatography (Gas Chromatograph 5890 Series II, Hewlett Packard Enterprise GmbH, Böblingen, Germany) as previously described by Koch et al. (2006). To determine the ammonia-N-concentration, a photometric measurement was performed as described before (Riede et al., 2013). Gas volume was measured daily using a gas drum type meter (Dr.-Ing. RITTER Apparatebau GmbH & Co. KG, Bochum, Germany). Samples for measuring of CO_2_ and CH_4_ were collected with a glass bulb at days 10, 14, 18, 22, 26, 30, 34, and 38. The analysis of the gas samples was performed at the ISAH (Institute for Sanitary Engineering and Waste Management, Leibniz University Hannover, Germany) by gas chromatography as described by Eger et al. (2018). The total production rate was calculated by multiplying the percentages of CO_2_ and CH_4_ with the gas volume corrected for standard conditions (1,013 hPa, 0°C). Samples for qPCR of the bacterial 16S gene in solid- and liquid-associated microorganisms (SAM and LAM, respectively) were collected at days 9, 16, 23, 30, and 37 by freezing feed bags or 10 mL of fermenter fluid, respectively, in liquid nitrogen. Samples were stored at −80°C until further treatment. Prior to DNA extraction, the samples were freeze-dried and the solid samples were ground mechanically using a coffee grinder. For sequencing and qPCR of the methyl coenzyme-M reductase gene (*mcrA*) gene, samples were taken weekly during BL and EP (days 10, 17, 24, 31, and 38). For the LAM, 30 mL of the liquid was taken from the vessel and treated as described by Eger et al. (2018). Feed bags were treated as described by Brede et al. (2020). Additionally, samples for metabolome analysis were obtained from the effluent on a weekly basis (days 10, 17, 24, 31, and 38) and stored at −18°C.

### DNA Extraction and Quantitative PCR

The DNeasy PowerSoil Kit (QIAGEN GmbH, Hilden, Germany) was used for DNA extraction in SAM and LAM samples as well as in qPCR samples to obtain the DNA for sequencing and qPCR analysis. Two modifications of the manufacture’s protocol were applied: 250 μL or 250 mg of samples collected for sequencing and *mcrA* PCR and 100 mg of the freeze-dried samples were used, and at the final step, the membrane was washed with 50 μL of 70°C pre-warmed water instead of solution C6. The concentration and purity of genomic DNA was determined using the NanoDrop ONE (Thermo Fisher Scientific Inc., Madison, WI, United States). A quantitative PCR was performed by Microsynth AG (Balgach, Switzerland) to determine the absolute abundance of bacteria as described by Liu et al. (2012). The quantification of methanogenic archaea based on the abundance of the *mcrA* gene was conducted at the Institute for Physiology and Cell Biology, University of Veterinary Medicine Hannover, Germany, by using primers against the *mcrA* gene as described by Denman et al. (2007). Reaction mixtures (20 μL) contained SensiFAST^TM^ SYBR^®^ No-ROX Kit (BioCat GmbH, Heidelberg, Germany), 500 nM of each primer and 10 ng template. The PCR product amplification was performed on a real-time PCR cycler (CFX96TM; Bio-Rad Laboratories Inc., Hercules, CA, United States) in accordance with the following protocol: 5 min at 95°C; 40 cycles of 15 s at 95°C; 30 s at 60°C; and 30 s at 72°C. In order to define the melt curve a thermal profile with a gradual increase in temperature (0.5°C/10 s) from 72 to 98°C was performed. Water was used as no-template control in each assay. Additionally, pool samples were calibrated with the NanoDrop ONE (Thermo Fisher Scientific Inc.) and were used to determine a standard curve (10^8^–10^2^ serial dilution). Each series of experiments was carried out twice. The efficiency of the PCR runs ranged between 88.6 and 90.3%.

### Next Generation Sequencing

Sequencing of the hypervariable regions V3 and V4 of the 16S RNA gene was performed by Microsynth AG on the Illumina MiSeq using a v2 500 cycles kit using Arch349F and Arch806R as primer pair for archaea (Takai and Horikoshi, 2000), and 341F_ill (5′-CCT ACG GGN GGC WGC AG-3′) and 802R_ill (5′-GAC TAC HVG GGT ATC TAA TCC-3′) for bacteria. Paired-ends reads were filtered through Illumina’s chastity filter, de-multiplexed and trimmed of Illumina adaptor residuals by Illumina real time analysis software. A quality check was performed with the software FastQC version 0.11.8. By using the software cutadapt v2.3, the locus specific V3 and V4 primers were cut from the sequencing reads. If trimming was not possible, the paired-end reads were discarded. The software USEARCH version 11.0.667 was used to unite the trimmed forward and reverse reads of each paired-end read. The merged sequences were filtered with the maximum of one “expected error” allowed. Reads containing ambiguous bases were discarded. Reads were denoised and OTUs (100% similarity) were compared to the SILVA v123 database as previously described (Eger et al., 2018). Alpha and beta diversity and the rarefaction were calculated by using the R package phyloseq v1.26. PERMANOVA was performed using the adonis2 function of vegan 2.5-7. The assessment of alpha diversity was conducted by using the Richness (Observed), Simpson and Shannon indices. The beta diversity calculation was performed with the unifrac distance measure. With the package DESeq2 v1.22.2 a differential OTU analysis based on normalized abundance counts was carried out and was considered significant at a log2fold change of at least ±2 and *P* < 0.05. In order to achieve a more specific classification, archaeal OTUs were compared to the JGI Integrated Microbial Genomes and Microbiomes (IGM/M) database^1^.

### Metabolome Analysis

The samples were analyzed at the Max Rubner-Institut (Department of Safety and Quality of Meat, Max Rubner-Institut, Federal Research Institute of Nutrition and Food, Kulmbach, Germany) by two-dimensional gas chromatography coupled with a quadrupole mass spectrometer (GC × GC qMS). The sample set consisted of biological study samples, quality check samples (QCs), and blank samples. The QC samples were prepared by combining an aliquot of 1 mL from each sample. Blank samples were matrix free. For sample preparation an aliquot of 1.5 mL of each sample was added to 20 μL of an internal standard mixture (Supplementary Table 2). Samples were centrifuged, filtered (0.2 μm), and freeze-dried. For the subsequent extraction, 200 μL of methanol (hypergrade LC/MS, Merck KGaA, Darmstadt, Germany) was added and shaken using the Bead Ruptor 24 Elite (Omni International Inc., Kennesaw GA, United States) for 30 s at 4 m/s. After a second centrifugation step (15 min at 4°C, 15,000 rpm) 150 μL of the supernatant was transferred into 2 mL glass vials containing a 200 μL glass insert, dried in a vacuum centrifuge (Christ Speedvac RVC 2-18 CDplus, Martin Christ Gefriertrocknungsanlagen GmbH, Osterode am Harz, Germany), and finally stored under protective argon atmosphere at −80°C until analysis. Before measurement, samples were derivatized by methoximation and silylation. Methoximation was performed using a 20 mg/mL solution of *O*-methoxylamine hydrochloride (Sigma-Aldrich, Darmstadt, Germany) in pyridine at 50°C for 1 h. In a second step, 50 μL of *N*-methyl-*N*-(trimethylsilyl)trifluoroacetamide + 1% trimethylchlorosilane (Thermo Fisher Scientific GmbH, Dreieich, Germany) was added and samples were shaken at 70°C for 1 h. Derivatized samples were additionally centrifuged and the clear supernatant was transferred to fresh glass vials. The measurements were performed on a Shimadzu GCMS QP2010 instrument (Shimadzu Deutschland GmbH, Duisburg, Germany). Instrumentation and parameter details are provided in Supplementary Table 3. Full scan data were acquired in a mass range of 60–550 m/z.

Peaks of the acquired raw data were integrated using the GCMS Postrun Analysis Module within the instrument software GCMSsolution (Version 4.45, Shimadzu Deutschland GmbH). Subsequently, peak quality filtering, peak alignment, signal intensity drift correction, and quality assessment were performed as described by Egert et al. (2015) and Weinert et al. (2015). For visualization of chromatograms and annotation of compounds, we used the NIST 14 library database implemented in GC Image Software (Version 2.7, GC Image, LLC, Lincoln, NE, United States). A series of *n*-alkanes (C7–C30, Sulpelco, Merck KGaA) was used as a retention time standard.

### Statistical Analysis

GraphPad Prism 8 (GraphPad Software Inc., San Diego, CA, United States) was applied for statistical analysis of biochemical data, qPCR results, and comparisons of relative abundances of microbial taxa. Residuals were tested for normal distribution by the Kolmogorov–Smirnov test. To determine time, treatment, and interaction (time × treatment) effects, a two-way ANOVA for repeated measurements and a *post hoc* Tukey test were performed. Due to missing values a mixed-effects analysis was carried out for pH value, α-diversity of solid-associated archaea and qPCR data of solid-associated bacteria. For qPCR data Tukey’s multiple comparison test was subsequently applied. Relative abundances of bacteria and archaea families were tested for normal distribution and compared with multiple *t*-tests corrected for multiple comparisons using Benjamini–Hochberg correction with an FDR of 1%. For all statistical test, the levels of significance were defined at ^∗^*P* < 0.05, ^∗∗^*P* < 0.01, ^∗∗∗^*P* < 0.001. Data are presented as mean ± SD. As no time-dependent alterations were observed during BL, only data for the last day (day 10) are presented.

For multivariate data analyses of metabolomics data unbiased principle component analysis (PCA) and supervised orthogonal projections to latent structures discriminant analysis (OPLS-DA) were applied using the SIMCA-P+ software (version 13, Umetrics, Umeå, Sweden). For univariate statistical testing the Tukey-HSD, which includes a correction for family-wise error rates by multiple testing, was applied using JMP software (13.1.0, SAS Institute Inc., Cary, NC, United States). The heatmap illustration of metabolite data was created in MetaboAnalyst^2^ (Chong et al., 2019).

### Data Availability

The sequence datasets are available at the NCBI SRA database under the accession number PRJNA716515.

## Results

### Production of Fermentation Gas

**FIGURE 1:**
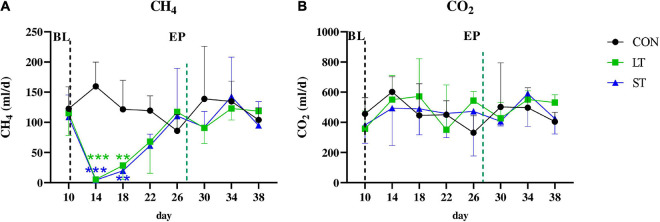
Daily production rates of methane **(A)** and carbon dioxide **(B)** during baseline period (BL, day 10) and experimental period (EP). The black line indicates the beginning of the EP. The green line indicates termination of Mootral supplementation in the short-term (ST) group. CON, control group; LT, long-term group. Significant differences between treatments and the control group in the Tukey *post hoc* test are indicated by ***P < 0.001, **P < 0.01. Data are presented as means ± SD.

Mootral treatment significantly affected methane production (*P* < 0.001, two-way ANOVA for repeated measurements, Figure 1A). The ANOVA also revealed a significant effect of time (*P* < 0.001) and an interaction of time × treatment (*P* < 0.01). In the post-test, a significant reduction in daily methane production compared to the CON group was observed at day 14 (*P* < 0.001, −97%) and day 18 (*P* < 0.01, −83% for ST, −77% for LT) for both groups treated with the feed additive. Subsequently, the production rate increased again and both groups did not differ from the CON group, indicating that this effect was not persistent. In contrast, CO_2_ production ranged from 331.43 ± 97.51 to 602.32 ± 87.20 mL in all three groups for the entire duration of the trial and did not differ among the three groups (*P* > 0.05, Figure 1B).

### Fermentation Parameters

Several changes in fermentation parameters were detected due to the treatment with Mootral. In the LT and ST group, the pH value was marginally lower compared to the CON group at some timepoints (Table 1, time: *P* < 0.001, treatment: *P* < 0.01, interaction: *P* < 0.01); however, all measured values were within the physiological range. An increase in NH_3_-N was observed in the LT group for the entire EP duration and in the ST within the supplementation time until day 27 (Table 1, time: *P* < 0.001, treatment: *P* < 0.001). This was accompanied by a slight increase in total SCFA production that was significant for the ST treatment at day 14 (*P* < 0.01) and for the LT treatment at days 22 and 26 (*P* < 0.01), as compared with the control (Table 1). Furthermore, Mootral treatment affected the SCFA composition. It led to a significant reduction in the molar proportion of acetate, in the ST group from day 14 to day 26 (*P* < 0.001) and at day 34 (*P* < 0.01), and in the LT group from day 14 to day 38 (*P* < 0.05) compared to CON group (Table 2). Propionate and butyrate proportions were significantly changed over time (*P* < 0.001, Table 2) with a slight increase in propionate and a transient elevation of butyrate. Moreover, a significant interaction of time and treatment (*P* < 0.01) was revealed for propionate and butyrate. Butyrate proportion was elevated in the LT group compared to the CON group at days 14, 18, and 26 (*P* < 0.05), and in the ST group at day 18 (*P* < 0.01). There were no significant differences among groups from day 26 onward. Additionally, the application of Mootral increased the valerate (*P* < 0.001) and isovalerate (*P* < 0.01) proportion (Table 2). Significant effects were also revealed for the factors time (both *P* < 0.001) and interaction (*P* < 0.01). The molar proportion of valerate was significantly higher in the LT group compared to the CON group (*P* < 0.05) from day 18 to day 38. In the ST group, the valerate proportion was increased compared to CON at days 22, 26, and 34 (*P* < 0.05). Regarding the isovalerate proportion the post-test revealed a significant increase at days 14–22 (*P* < 0.001) in the LT and ST group compared to the CON group.

**TABLE 1:** Fermentation parameters at day 10 (baseline period) and every fourth day of experimental period (days 14–38).

**Parameter**	**Day**	**Treatment**			**Pooled SD**	***P*-value**		
		**CON**	**LT**	**ST**		**Treatment**	**Time**	**Interaction**
pH	10	6.77	6.80	6.81	0.02	0.007	<0.001	0.009
	14	6.77^a^	6.66^b^	6.66^ab^	0.03			
	18	6.76^a^	6.70^a^	6.69^b^	0.02			
	22	6.81	6.76	6.76	0.01			
	26	6.77	6.72	6.73	0.01			
	30	6.79	6.95	6.8	0.03			
	34	6.79	6.74	6.79	0.02			
	38	6.82	6.78	6.81	0.02			
NH_3_-N (mM)	10	6.77	7.07	6.55	0.17	<0.001	<0.001	<0.001
	14	7.12^a^	9.05^b^	9.77^b^	0.42			
	18	6.71^a^	9.75^b^	9.97^b^	0.26			
	22	6.28^a^	9.17^b^	9.56^b^	0.29			
	26	6.66^a^	9.42^b^	9.87^b^	0.40			
	30	6.82^a^	9.61^b^	7.52^a^	0.12			
	34	6.74^a^	8.62^b^	7.65^a^	0.31			
	38	6.63^a^	8.70^b^	7.26^a^	0.57			
SCFA total (mmol/day)	10	27.62	26.8	24.83	1.39	0.185	<0.001	0.005
	14	29.11^a^	33.59^ab^	37.05^b^	3.07			
	18	27.31	33.4	32.98	1.67			
	22	23.76^a^	30.73^b^	28.07^ab^	1.77			
	26	24.87^a^	31.91^b^	30.65^ab^	2.23			
	30	26.13	26.70	23.13	1.65			
	34	26.29	29.37	27.08	1.49			
	38	22.42	27.75	21.60	1.65			

*ST, short-term group; LT, long-term group; CON, control group. Data are presented as means and pooled SD. Statistical analysis was performed by repeated measurements two-way ANOVA (NH_3_-N and SCFA) or mixed-effects analysis (pH), followed by Tukey *post hoc* test. Significant differences within a row are indicated by different superscripts.*

**TABLE 2:** Molar proportions of acetate, propionate, butyrate, valerate, isovalerate at day 10 (baseline period), and every fourth day of experimental period (days 14–38).

**Parameter**	**Day**	**Treatment**			**Pooled SD**	***P*-value**		
		**CON**	**LT**	**ST**		**Treatment**	**Time**	**Interaction**
Acetate (%)	10	58.23	57.69	59.77	0.52	<0.001	<0.001	<0.001
	14	58.62^a^	48.66^b^	47.98^b^	0.79			
	18	57.59^a^	48.48^b^	49.40^b^	0.72			
	22	59.87^a^	51.21^b^	50.51^b^	0.88			
	26	59.74^a^	51.94^b^	54.50^b^	1.18			
	30	57.84^a^	54.89^b^	56.98^ab^	0.42			
	34	59.34^a^	54.5^b^	56.08^b^	0.80			
	38	58.32^a^	53.48^b^	57.15^a^	1.33			
Propionate (%)	10	31.43	31.76	31.33	0.47	0.095	<0.001	<0.001
	14	32.19^a^	32.85^a^	35.63^b^	0.91			
	18	34.09	34.02	33.92	0.71			
	22	32.12^a^	33.41^ab^	34.97^b^	1.14			
	26	33.03	35.43	35.14	1.03			
	30	34.35	34.94	34.35	0.85			
	34	32.05^a^	36.27^b^	34.55^ab^	0.65			
	38	32.9^a^	37.64^b^	34.24^a^	0.76			
Butyrate (%)	10	7.96	7.12	6.26	0.74	0.091	<0.001	0.005
	14	7.84^a^	10.88^b^	9.59^ab^	0.86			
	18	7.99^a^	11.14^b^	11.20^b^	0.64			
	22	7.80^a^	9.89^ab^	10.19^b^	0.61			
	26	7.02^a^	9.59^b^	8.59^ab^	0.68			
	30	7.43	8.99	7.70	0.63			
	34	7.76	8.59	7.76	0.47			
	38	8.15	8.18	7.06	0.78			
Valerate (%)	10	0	0	0	0	<0.001	<0.001	0.004
	14	0	0.15	0	0.08			
	18	0^a^	0.80^b^	0.21^a^	0.12			
	22	0^a^	0.83^b^	0.77^b^	0.26			
	26	0^a^	1.32^b^	0.78^b^	0.26			
	30	0^a^	0.96^b^	0.22^a^	0.15			
	34	0^a^	0.65^b^	0.71^b^	0.20			
	38	0^a^	0.71^b^	0.54^ab^	0.24			
Isovalerate (%)	10	2.37	3.43	2.65	0.45	0.004	<0.001	<0.001
	14	1.35^a^	7.46^b^	6.8^b^	0.45			
	18	0.32^a^	5.56^b^	5.27^b^	0.82			
	22	0.21^a^	4.66^b^	3.56^b^	0.54			
	26	0.20	1.72	0.98	0.32			
	30	0.38	0.22	0.74	0.60			
	34	0.85	0	0.91	0.52			
	38	0.62	0	1.02	0.45			

*ST, short-term group; LT, long-term group; CON, control group. Data are presented as means and pooled SD. Significant differences within a row are indicated by different superscripts.*

### Effects of Mootral on Abundance of 16S rRNA Genes and Bacterial Community Composition

The abundance of the bacterial 16S rRNA gene was not altered by Mootral treatment (data not shown). By sequencing the bacterial 16S gene V3 and V4 regions, 1,031 OTUs were detected in LAM samples, 1,026 of these were bacterial OTUs. Of the 749 observed solid-associated OTUs, 744 were bacterial OTUs. Alpha-diversity measures did not differ among treatment groups (Supplementary Figure 1). In total 18 phyla were detected, the most abundant being Bacteroidetes (LAM: 40.24 ± 6%; SAM: 25.2 ± 3.5%) and Firmicutes (LAM: 31.55 ± 3.35%; SAM: 57.03 ± 6.45%). A total of 43 bacterial families were detected in the LAM samples and 39 families in the SAM samples. Only the 20 most abundant thereof will be discussed below (Supplementary Figure 2). Few differences among treatment groups were detected by multiple comparisons (Table 3). A lower abundance of the families Christensenellaceae and Succinivibrionaceae was observed in the LAM samples at day 17 in the LT group compared to the CON group (CON vs. LT, *P* < 0.001). At days 31 and 38 of the experiment, the relative abundance of Prevotellaceae was higher in the LT group compared to the CON group (CON vs. LT, *P* < 0.001). In the SAM samples, only the family Victivallaceae was less abundant in the LT group at day 17 (CON vs. LT, *P* < 0.001). Lactobacillaceae were numerically more abundant in the ST group at days 17 and 24, and in the LT group at days 31 and 38, however, due to a high variance within the treatments this effect was not significant.

**TABLE 3:** Differentially abundant bacterial families by treatment group in liquid- and solid-associated microbiota (LAM and SAM, respectively).

	**Day**	**Treatment**			**Pooled SD**
		**CON**	**LT**	**ST**	
LAM					
Christensenellaceae	10	0.37	0.30	0.43	0.14
	17	0.64^a^	0.30^b^	0.31^b^	0.06
	24	0.73	0.57	0.50	0.10
	31	1.05	0.62	0.70	0.29
	38	1.10	0.82	0.75	0.29
Prevotellaceae	10	16.4	15.1	15.9	3.47
	17	13.5	17.4	20.5	3.01
	24	13.8	17.2	17.8	2.44
	31	20.3^a^	31.7^b^	18.2^ab^	1.76
	38	15.1^a^	24.2^b^	15.0^a^	1.76
Succinivibrionaceae	10	13.71	11.44	12.34	4.31
	17	12.32^a^	4.01^ab^	3.02^b^	2.30
	24	11.53	8.45	8.76	5.37
	31	8.69	1.64	4.50	2.35
	38	8.69	1.64	4.50	2.35
SAM					
Victivallaceae	10	0.59	0.59	0.69	0.14
	17	0.58^a^	0.16^b^	0.17^ab^	0.09
	24	0.84	0.39	0.33	0.16
	31	0.86	0.17	0.53	0.25
	38	0.86	0.23	0.82	0.15

*ST, short-term group; LT, long-term group; CON, control group. Data are mean relative abundances (%) and pooled SD. Families that differed significantly by multiple *t*-test (*P* < 0.05, Benjamini–Hochberg correction, FDR = 1%). Different superscript indicate significant differences within a row.*

### Effects of Mootral on *mcrA* Copy Numbers and on the Archaeal Community

**FIGURE 2:**
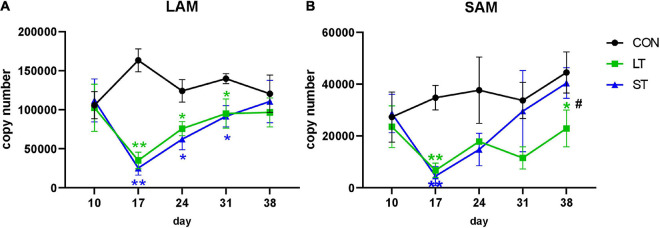
Copy numbers of the *mcrA* gene in LAM **(A)** and SAM **(B)** samples. ST, short-term group; LT, long-term group; CON, control group. Significant treatment effects of LT or ST, compared to CON in Tukey *post hoc* test are indicated by **P < 0.01, *P < 0.05. Significant treatment effects between ST and LT are indicated by ^#^*P* < 0.05. Data are presented as means ± SD.

In contrast, *mcrA* copy numbers (Figure 2) were affected by time (SAM: *P* < 0.001; LAM: *P* < 0.01), treatment (both *P* < 0.001) and an interaction of both factors (LAM: *P* < 0.001; SAM: *P* < 0.01). Mootral treatment led to a significant decrease in *mcrA* gene copy number at day 17 (*P* < 0.01) in both LAM and SAM (Figures 2A,B). In LAM (Figure 2A), a reduced copy number was also observed at days 24 and 31 (*P* < 0.01). In SAM, *mcrA* gene copy numbers were significantly lower in LT group compared to both the ST and CON groups, at day 38 (*P* < 0.05, Figure 2B).

In the liquid samples, archaea sequencing resulted in 2,630,324 total reads, ranging from 270 to 810,506 with a mean of 37,046. Within these, 71 OTUs were identified, 42 of them belonged to the domain *Archaea* and accounted for 92.9% of the reads. In the solid samples, a total of 2,279,860 reads were obtained, the range was from 2,147 to 105,637 with a mean of 38,642 among all samples. In solid samples, 97.0% of the sequencing reads belonged to *Archaea* and 40 of the detected 54 OTUs were archaeal OTUs. All archaeal OTUs belonged to the phylum Euryarchaeota. The three classes Methanomicrobia, Thermoplasmata, and Methanobacteria were identified.

**FIGURE 3:**
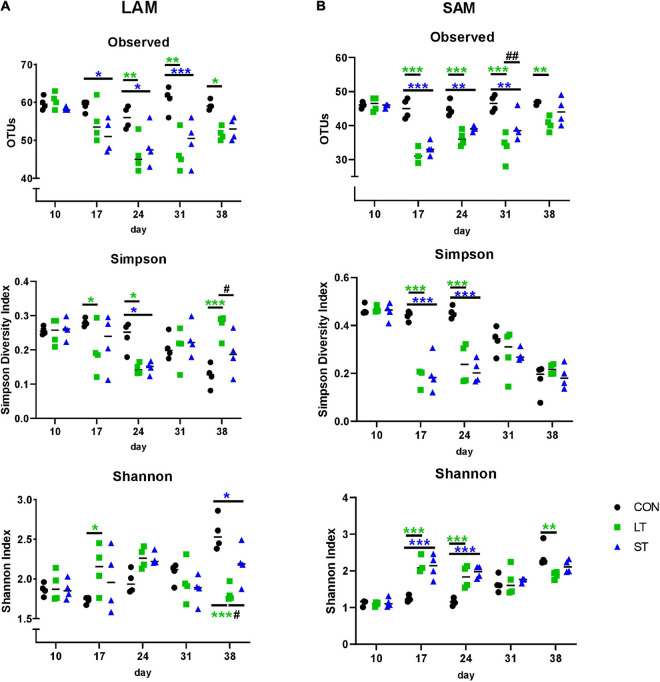
Number of observed operational taxonomic units (OTUs), Simpson’s Diversity Index, and Shannon’s Index in LAM **(A)** and SAM **(B)** of *Archaea* by treatment groups (*n* = 4). Individual data and medians are presented. ST, short-term group; LT, long-term group; CON, control group. The treatment effects of the Tukey *post hoc* test are indicated by *P < 0.05, **P < 0.01, ***P < 0.001 (CON vs. LT or CON vs. ST); ^#^*P* < 0.05, ^##^*P* < 0.01 (LT vs. ST). Time effects are not shown.

**FIGURE 4:**
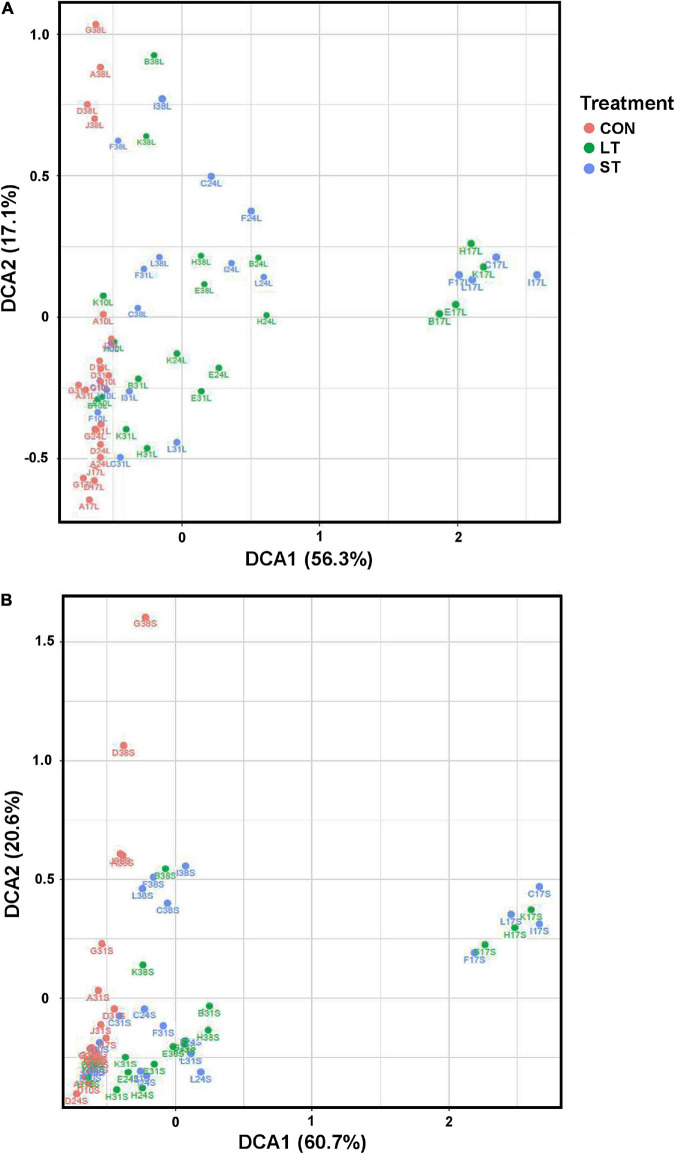
The beta-diversity of *Archaea* is indicated by Detrending Correspondence Analysis (DCA) based on the UniFrac distances. Treatment groups are distinguished by color: CON, red; LT, green; ST, blue. The labels represent the day (10, 17, 24, 31, and 38) and the name of the fermentation vessel (A–L). Panel **(A)** shows the LAM, and **(B)** shows the SAM archaeal communities.

Mootral application also changed archaeal alpha-diversity measures. In both, LAM and SAM samples, the number of observed OTUs decreased up until day 31 in ST group and up until day 38 in the LT group (*P* < 0.05, Figures 3A,B). Moreover the Simpson Diversity index decreased for the LT and ST groups at days 17 and 24 in SAM (*P* < 0.001, Figure 3B), for LT at days 17, 24, and 38 in LAM (*P* < 0.05, Figure 3A), as well as for ST at day 24 in LAM (*P* < 0.05). The Shannon index was affected at days 14, 24, and 38 in SAM (*P* < 0.01, Figure 3B), but only at days 17 and 38 in LAM (*P* < 0.05, Figure 3A). In the beta-diversity plots of archaea, all 12 communities clustered close together in both liquid and solid samples at day 10 (Figure 4). However, at day 17 the LT and ST communities were clearly separated from the day 17 CON samples as well as from the day 10 samples. At day 24 and day 31, the LT and ST samples were widespread in the plot, whereas the two groups treated with Mootral moved closer to CON group at day 38 but remained more variable than the samples of the CON group. PERMANOVA revealed a clear effect of time and interaction for both phases (*P* < 0.001), as well as a treatment effect for LAM (*P* < 0.05) and SAM (*P* < 0.01).

At day 10, the Methanomicrobiaceae was the family with the highest abundance in all three treatment groups (LAM: 61.7 ± 5.1%; SAM: 90.9 ± 1.9%, Table 4). Additionally, the families Thermoplasmatales incertae sedis (LAM: 35.9 ± 4.8%; SAM: 8.7 ± 1.7%), Methanobacteriaceae (LAM: 2.3 ± 0.5%; SAM: 0.6 ± 0.3%), and Methanosarcinaceae (LAM: 0.008 ± 0.08%; SAM: 0.001 ± 0.001%) were detected. After treatment with Mootral Thermoplasmatales incertae sedis increased in the LT group and ST group. In contrast, a significant reduction in the proportion of Methanomicrobiaceae was observed (Table 4).

**TABLE 4:** Differentially abundant archaeal families by treatment group in liquid- and solid-associated microbiota (LAM and SAM, respectively).

	**Day**	**Treatment**			**Pooled SD**
		**CON**	**LT**	**ST**	
LAM					
Methanobacteriaceae	10	2.18	2.42	2.36	0.52
	17	1.51	3.25	1.63	1.32
	24	1.43^a^	8.80^ab^	14.29^b^	4.02
	31	1.87	3.39	1.05	1.77
	38	7.99	7.00	2.74	3.00
Methanomicrobiaceae	10	60.42	61.04	63.74	5.60
	17	66.68^a^	8.24^b^	4.94^b^	3.22
	24	61.36^a^	34.54^ab^	16.36^b^	9.88
	31	54.57	48.16	55.82	15.44
	38	16.86	14.60	27.87	9.80
Methanosarcinaceae	10	0.01	0.01	0.01	0.01
	17	0.01^a^	0.07^b^	0.10^ab^	0.07
	24	0.16	0.03	3.97E−03	0.10
	31	0.35	2.20E−03	2.46E−03	0.19
	38	1.39	1.66E−03	0.06	0.59
Thermoplasmatales incertae sedis	10	37.39	36.53	33.89	5.24
	17	31.80^a^	88.44^b^	93.33^b^	3.99
	24	37.06^a^	56.63^ab^	69.35^b^	11.79
	31	43.21	48.44	43.13	15.08
	38	73.76	78.40	69.33	9.63
SAM					
Methanobacteriaceae	10	0.74	0.52	0.63	0.28
	17	0.70^a^	2.24^b^	3.22	1.78
	24	1.64	7.55	10.30	4.06
	31	1.28	6.24	1.75	2.73
	38	6.07	9.21	2.03	4.65
Methanomicrobiaceae	10	90.99	91.34	90.26	2.61
	17	90.02^a^	8.42^b^	10.86^b^	4.43
	24	90.24^a^	62.86^ab^	54.98^b^	11.93
	31	77.91	73.19	72.83	9.59
	38	37.78	38.83	41.96	15.51
Methanosarcinaceae	10	2.13E−03	2.79E−04	1.52E−03	8.45E−04
	17	2.35E−03	0.06	0.05	0.09
	24	0.01	0	2.10E−03	0.01
	31	0.06	0	3.42E−03	0.04
	38	0.25	4.69E−03	0.01	0.09
Thermoplasmatales incertae sedis	10	8.27	8.14	9.11	1.92
	17	9.28^a^	89.28^b^	85.87^b^	6.13
	24	8.11^a^	29.59^ab^	34.72^b^	9.59
	31	20.76	20.57	25.42	7.17
	38	55.90	51.96	56.00	12.49

*ST, short-term group; LT, long-term group; CON, control group. Data are mean relative abundances (%) and pooled SD. Families that differed significantly by multiple *t*-test (*P* < 0.05, Benjamini–Hochberg correction, FDR = 1%). Different superscript indicate significant differences within a row.*

**FIGURE 5:**
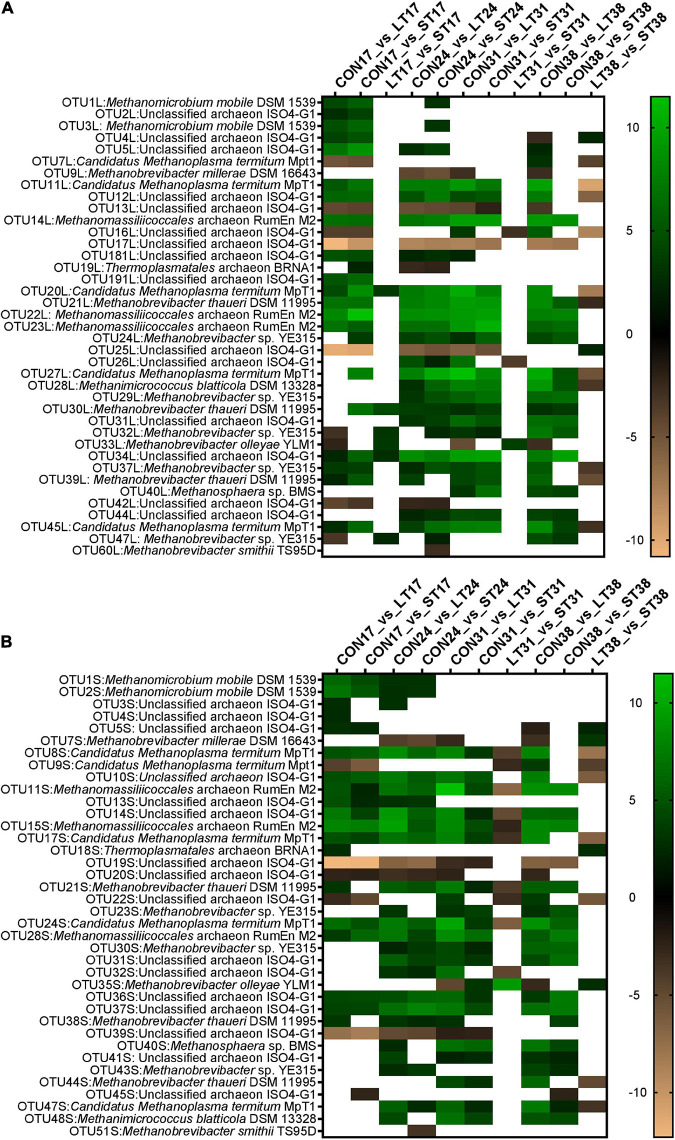
Heatmaps of significantly differentially abundant (*P* < 0.05, differential OTU analysis, DESeq2) archaeal operational taxonomic units (OTUs) in liquid-associated **(A)** and solid-associated **(B)** microbiota based on log2fold changes, which is indicated by the brown to green color scale. A threshold of ±2 was applied, and white indicate no significant differences. The heatmap displays the significant differences at days 17, 24, 31, and 38 between the three treatment groups (CON, LT, and ST). No significant differences were detected at day 10 and at day 24 between LT and ST group.

The analysis of differentially abundant OTUs revealed significant alterations in liquid (Figure 5A) and solid (Figure 5B) archaeal communities among treatment groups and at different time points. None of the OTUs differed among the groups at day 10, before treatments were applied. Mootral application diminished the abundance of members of the genera *Methanomicrobium* (*Methanomicrobium mobile* DSM 1539), *Methanobrevibacter* (*Methanobrevibacter thaueri* DSM 11995, *Methanobrevibacter* sp. YE 315), and the family Thermoplasmatales incertae sedis (Unclassified archaeon ISO4-G1, *Candidatus Methanoplasma termitum* MpT1 and *Methanomassiliicoccales* archaeon RumEn M2) at days 17 and 24 in SAM and LAM (Figure 5), while it increased the relative abundance of several OTUs from Thermoplasmatales incertae sedis. In the LT group, *Methanobrevibacter olleyae* YLM1 was enriched at several time-points in both phases. While LT and ST samples were similar at day 24 for both phases, at day 31, *M. thaueri* DSM 11995 and nine OTUs from Thermoplasmatales incertae sedis increased again in ST SAM samples. In LAM, 12 OTUs displayed consistent differences among the CON and LT groups throughout the entire treatment period. Five of these 12 OTUs also differed between the ST and CON group up until day 31, but not at day 38.

### Effects of Mootral on the Rumen Metabolome

**FIGURE 6:**
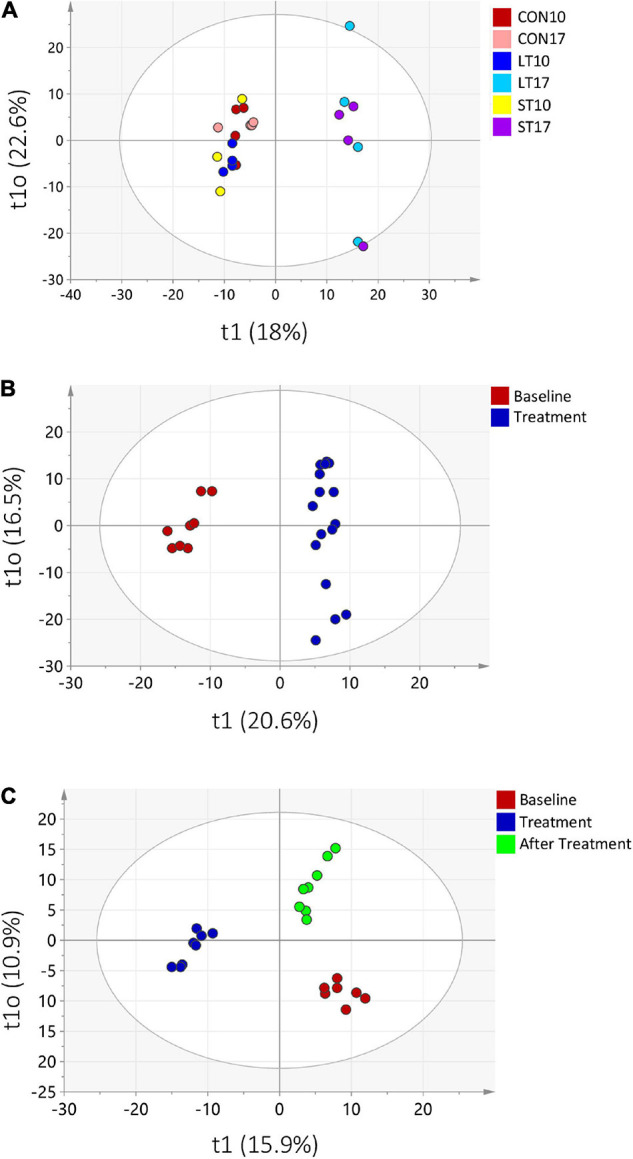
Orthogonal projections to latent structures discriminant analysis plot of the metabolomics analysis **(A)** for all three treatment groups at days 10 and 17, **(B)** the long term (LT) group at days 17–38 (Treatment) vs. LT and CON at day 10 (BL) and **(C)** the short term (ST) group at days 17 and 24 (Treatment), at days 31 and 38 (After Treatment) vs. CON and ST at day 10 (BL).

Metabolite profiles of the samples were compared with focus being placed on differences among treatments (comparison among BL, and CON, ST, and LT groups at day 17, and time-dependent shifts in the ST and LT groups). Addition of Mootral lead to a clear separation of ST and LT samples at day 17, from BL samples at day 10 in PCA and OPLS-DA (Supplementary Figure 3A, Figure 6A, *R*^2^ = 0.97 and *Q*^2^ = 0.88). For the LT group, a clear separation of day 10 CON and LT samples vs. LT samples from day 17 to day 38 (Mootral addition) was confirmed (*R*^2^ = 0.98, *Q*^2^ = 0.94, Figure 6B). In contrast, in the ST group, a model with three groups was applied, comparing “before treatment” samples from day 10 (BL, CON10, and ST10), with Mootral treatment (ST17 and ST24) and “after treatment” samples (ST31 and ST38). Both PCA and OPLS-DA (*R*^2^ = 0.91, *Q*^2^ = 0.77, Figure 6C and Supplementary Figure 3C) indicated clear differentiation between pre-treated and treated samples. Samples collected after treatment were not clearly separated from day 10 samples in PCA (Supplementary Figure 3C) and in the OPLS-DA they formed a third cluster (Figure 6C). To determine compounds which were responsible for the observed clustering in the OPLS-DA model, variable importance in projection (VIP) score was calculated. A threshold of VIP >1 was chosen, as this indicated an above average contribution of this compound for the model. For the LT group, 194 compounds were significantly different (*P* < 0.05) and exhibited a VIP score >1; for ST group, 222 compounds differed. About 38% (106) of these compounds could be identified. The 20 identified compounds with the highest VIP scores for both comparisons (ST and LT) were selected (Supplementary Table 4). Four clusters were identified (Supplementary Figure 4). The first cluster was more highly concentrated when the samples were treated with Mootral compared to BL (day 10), ST31 and ST38 samples and could therefore contain compounds derived from Mootral itself or degradation products of Mootral. The second cluster was enriched in BL samples and was persistently reduced by Mootral application. It consisted of propylene glycol, two branched-chain SCFA, 2-hydroxycaproic acid, and two monosaccharides. A third group consisting mainly of plant-derived compounds showed a similar pattern; however, a slight recovery of these compounds appeared in ST31 and ST38. The fourth cluster exhibited a suppression during Mootral treatment, but clearly recovered after terminating the supplementation in ST group (ST31 and ST38). This cluster consisted of several fatty acids, phenoxyethanol, 5-hydroxytryptophan, 4-isopropylphenol, and an indole derivate.

## Discussion

The strong potential of Mootral, a product consisting of garlic and citrus extracts, to reduce methane emissions has been previously revealed *in vitro* and *in vivo* (Eger et al., 2018; Roque et al., 2019; Vrancken et al., 2019). The aim of the present study was to examine the long-term effects of Mootral on ruminal fermentation parameters, archaeal, and bacterial community structure as well as on the rumen metabolome in a long-term *in vitro* trial.

### Mootral Dosage and Potential Effects of Mootral Degradation

Due to the use of natural compounds in this product, the concentrations of active substances varies. As a reference, the concentration of allicin was determined. The dose applied here resulted in an allicin concentration of approximately 20 mg/L in the fermentation vessels, which is comparable to doses applied in previous *in vitro* experiments (Busquet et al., 2005; Chaves et al., 2008; Eger et al., 2018) and far lower than the likely toxicity for rumen bacteria (300–3,000 mg/L) (Busquet et al., 2005). In general, the dosage applied in the RUSITEC system needs to be higher compared to *in vivo* dosing due to the higher liquid to solid ratio (Carro et al., 2009), however, also *in vivo* quite high dosages (2 g allicin/65 kg body weight) have been used (Ma et al., 2016).

Due to a low allicin concentration in the Mootral powder used in this study, a relatively large amount had to be used. As Mootral is a natural product with various active compounds, an inactive placebo for the control group was not available. Therefore, it cannot be excluded, that some of the effects observed in this study are due to nutrient utilization of the product. Metabolites, whose concentrations were increased at the same timepoints when Mootral was applied are probably derived from the product itself. As the crude protein content of the powder is about 22% (Supplementary Table 1), elevated NH_3_-N levels are presumably related to the higher protein supply. Mootral application also increased the concentration of benzeneprenoic acid (hydrocinnamic acid) and other aromatic compounds. Hydrocinnamic acid is produced by rumen bacteria from cinnamic acid (Martin, 1982) which can be found in various plants. Bitter oranges not only contain isoflavonoids such as naringin, but, e.g., also phenolic acids, hydroxybenzoic acid, and cinnamic-acid (Jabri Karoui and Marzouk, 2013), therefore it is most likely that the increase in the concentrations of aromatic compounds is based on supply by Mootral application. The concentration of pyridoxine, a vitamin B6, was also increased during Mootral application. In contrast to other B vitamins its rumen balance is often negative (Castagnino et al., 2016; Beaudet et al., 2020) and an increase in its concentration may be beneficial for the animal.

### Effects of Mootral on the Fermentation Pattern, the Bacterial Community Composition and Metabolites in the Rumen Simulation Technique System

In the present trial, the supplementation of Mootral induced an increase in the total SCFA production in the RUSITEC-system. This was due to an increased production of propionate, butyrate, valerate, and isovalerate. However, this effect was transient for total SCFA, butyrate, and isovalerate even in the LT group. Effects on pH, SCFA production and SCFA molar proportions resembled those observed by Eger et al. (2018) and Ahmed et al. (2021). Previous studies with garlic compounds and flavonoids are variable, but they often also report an increase in total SCFA accompanied by a lower acetate, and higher propionate or butyrate proportions (Busquet et al., 2005; Balcells et al., 2012; Patra and Yu, 2015; Ma et al., 2016). Metabolomics revealed that Mootral supplementation suppressed the production of several fatty acids (short, medium, and long chained), phenol derivates, and plant-derived compounds. Changes in carbohydrate metabolism were indicated by accumulation of a disaccharide and suppression of several monosaccharides, levoglucosan, and tyrosol.

The abundance of bacteria was not affected by Mootral application, which is in agreement with previous reports where garlic oil (Patra and Yu, 2015) or the flavonoid naringin (Oskoueian et al., 2013) were investigated *in vitro* using rumen fluid from dairy cattle (roughage: concentrate 43:57) or male cattle (roughage: concentrate 60:40), respectively. In contrast, *in vitro* treatment with garlic powder resulted in a dose-dependent reduction in the number of bacteria with a straw and concentrate diet (Wanapat et al., 2008). Treatment with garlic oil led to a reduction in the total number of the cellulolytic bacteria *Fibrobacter succinogenes*, *Ruminococcus flavefaciens*, and *Ruminococcus albus* (Patra and Yu, 2012). Cellulolytic bacteria mainly produce acetate (Dijkstra et al., 2012), which was lower in proportions in Mootral-treated groups in the present study and is a source of hydrogen for methane production. However, only the families Succinivibrionaceae, Prevotellaceae, Christensenellaceae, and Victivallaceae were affected at single time-points by Mootral treatment in our study. Ahmed et al. (2021) also observed a treatment related effect on the family Prevotellaceae, as the relative abundance was significantly increased with a treatment of 20% Mootral. Nevertheless, they only observed additional effects for Veillonellaceae. Altogether, the impact of Mootral on the bacterial community appears to be small and was only visible at single time-points. However, most rumen bacteria have a certain metabolic flexibility to degrade various substrates (Comtet-Marre et al., 2017) and therefore may result in differing fermentation profiles. Thus, transient shifts in fermentation products may occur without major alterations in the bacterial community composition. As the shifts in methane production were not in parallel to shifts in fermentation products, the effects of Mootral on methane production are not simply linked to the changing bacterial fermentation pattern.

### Effects of Mootral on Methane Production and the Archaeal Community

Regarding their ability to reduce methane production, both garlic and flavonoids have been described as very effective (Busquet et al., 2005; Patra et al., 2011; Soliva et al., 2011; Ramos-Morales et al., 2018). Compared to this study, a similar effect on methane production was observed in the previous *in vitro* study by Eger et al. (2018), in which a 95% reduction in methane production was achieved during an 8 day supplementation period. The reduction in *in vivo* methane production in beef cattle or dairy cattle by Mootral was considerably less, ranging from 21 to 38% (Roque et al., 2019; Vrancken et al., 2019). In the *in vitro* study by Ahmed et al. (2021), a reduction in the percentage of methane in the fermentation gas by 54% could be achieved in a batch culture system when 20% Mootral was administered using rumen fluid from sheep fed grass and concentrate (50:50). However, the total production rate in the supplemented group did not differ from the control group (Ahmed et al., 2021). A discrepancy between the decrease in methane production achieved *in vivo* compared to *in vitro* was also observed by Fievez et al. (2003) when comparing the effects of different fish oil types in a batch culture incubation with an *in vivo* application. An inhibition of rumen methanogenesis was observed in both approaches, but the effect was one-fifth *in vivo*. It appears that the methane-reducing effect of Mootral is reproducible in different animals and with different diets and *in vitro* systems. However, as our long-term study indicates, this effect appears to be transient, at least *in vitro*, as methane production was not significantly reduced after 12 days of supplementation. Long-term responses to different methane reducing compounds differ. Long-term application may result in a better and more persistent decrease in methane production (Belanche et al., 2020) or reveal a transient effect (Guan et al., 2006). Patra et al. (2011) did not observe an effect of garlic bulb on methane emissions of sheep after 21 days of feeding. Schilde et al. (2021) revealed that 3-NOP combined with a high concentrate ration *in vivo* persistently reduced methane production, while when a high forage ration was fed, methane production recovered after 3–4 months. In contrast to the short suppression of methane production in our study, the two *in vivo* studies using Mootral (Roque et al., 2019; Vrancken et al., 2019) detected effects over a 12-week supplementation period; however, these two studies used higher concentrate proportions compared to our *in vitro* study. Therefore, further studies on the time-course of methane emissions and ruminal parameters in response to Mootral supplementation in combination with different feeding strategies *in vivo* and *in vitro* are needed to reveal whether the transient effects observed here are linked to the *in vitro* system or to the dietary strategy and whether they transfer to the *in vivo* situation.

Busquet et al. (2005) discussed that the mechanism of methane inhibition by organosulfur components in garlic could be based on an inhibition of the 3-hydroxy-3-methyl-glutaryl coenzyme A, which is needed to produce membrane lipids only found in archaea, however, they could not clearly prove or reject this hypothesis. However, this agrees with previous reports on allicin inhibiting enzymes by reacting with their thiol group (Ankri and Mirelman, 1999). Alterations to the liquid-associated archaeal communities have already been reported by Eger et al. (2018) where a similar diet was used in the RUSITEC system. In the present study solid-associated archaea were additionally investigated. The qPCR targeting the *mcrA* gene revealed a significant reduction in methanogenic archaea, which is in line with previous studies on garlic or flavonoids (Oskoueian et al., 2013; Patra and Yu, 2015; Ma et al., 2016). However, this effect was not consistent over time indicating an adaption of these microorganisms to the treatment. In addition, the application of Mootral resulted in a reduction in the relative abundance of Methanomicrobiaceae and an increase in *Methanomassiliicoccales*. This is in line with the finding of Ahmed et al. (2021), who observed a dose-dependent increase in the *Methanomassiliicoccaceae*. Interestingly, in the previous studies by Eger et al. (2018), *Methanobrevibacteriaceae* dominated before treatment and were reduced by Mootral application; however, this resulted in a comparable decrease in methane reduction. Nonetheless, as with methane production, the Mootral-induced structural changes in the microbial community composition were transient, indicating an adaptation to the product. The ability of the microbial ecosystem to adapt to dietary strategies to reduce methanogenesis is well known (Beauchemin, 2009). This effect might not have occurred in the *in vitro* study by Ahmed et al. (2021), as a short-term batch culture was used, whereas in our trial, the supplementation lasted for up to 28 days. Saro et al. (2018) reported that feeding growing lambs with a combination of garlic and linseed oil for 4 weeks did not result in persisting methane reduction or changes of the archaeal population.

Although the composition of the ST and LT archaeal communities was similar to the CON group by the end of the experiment, the log2fold change revealed an increased abundance of some OTUs in the treated groups. This was particularly applicable to OTUs belonging to *Methanomassiliicoccales*. When this analysis was performed these OTUs were still classified as Thermoplasmatales incertae sedis in the database, however, nowadays their classification has to be updated. Most *Methanomassiliicoccales* OTUs were identified as ISO4-G1 or RumEn M2, which are now classified to belong to the host-associated clade *Methanomethylophilaceae* (Söllinger et al., 2016; Cozannet et al., 2020). It has been demonstrated that they produce methane in a H_2_-dependent methylotrophic way and that their reduction is accompanied by a reduced methane production (Borrel et al., 2013; Poulsen et al., 2013). In contrast, in the present study, an increase in the proportion of *Methanomassiliicoccales* and decrease in the abundance of Methanomicrobiaceae accompanied the reduction in methane production. However, differing results for several OTUs that appeared to be closely related indicate that *Methanomassiliicoccales* have a certain metabolic flexibility. Methyl-reducing archaea such as *Methanomassiliicoccus luminyensis* have been demonstrated to be able to use lower H_2_ pressures compared to “classic hydrogenotrophic” (growing on CO_2_ + H_2_) such as *Methanobrevibacter* strains (Feldewert et al., 2020). One potential mode of action of Mootral could therefore be to provide an alternative sink for H_2_, thereby favoring obligate methylotrophic archaea. Calculated based on SCFA, hydrogen production was not reduced by Mootral addition, raising the question of the fate of the hydrogen (Eger et al., 2018). Moreover, a higher availability of methyl-compounds through Mootral addition may favor these archaea. The results of Eger et al. (2018) implied that the effect of Mootral is primarily mediated *via* a reduction in *Methanobrevibacter* spp. of the SGMT clade. In the present study, we also revealed a suppression of *M. thaueri* and an additional suppression of *Methanomicrobium mobile*, while an enrichment of *M. olleyae* which belong to the lower-methane production RO-clade (Danielsson et al., 2012) was observed in the LT group. Some of these shifts persisted, while the total archaeal family composition resembled the CON group at the end of the experiment. To our knowledge, there are currently no data on microbial community composition available *in vivo*, and it has to be investigated whether these shifts are mirrored and persistent *in vivo*.

## Conclusion

By using a long-term *in vitro*-system, we demonstrated that the methane-reducing effect of Mootral is mainly based on direct effects on the liquid and solid-associated archaea. Although some changes in fermentation products were observed, the bacterial community was barely affected. As most of the effects were transient even with continued supplementation, it remains to be studied, whether this effect also occurs *in vivo* and under different feeding strategies or if it is related to the *in vitro* system in order to find a practicable application scheme for the on-farm use of Mootral.

## Data Availability Statement

The datasets presented in this study can be found in online repositories. The names of the repository/repositories and accession number(s) can be found below: https://www.ncbi.nlm.nih.gov/, PRJNA716515.

## Ethics Statement

The animal study was reviewed and approved by the Lower Saxony State Office for Consumer Protection and Food Safety Oldenburg, Germany AZ 33.19-42502-05-13A373.

## Author Contributions

GB and MB designed the experiments. JB, MB, and MP were responsible for data acquisition and statistical analysis. BE processed the metabolomics data. JB and MB wrote the manuscript. All authors approved the final version of the manuscript.

## Conflict of Interest

The authors declare that the research was conducted in the absence of any commercial or financial relationships that could be construed as a potential conflict of interest.

## Publisher’s Note

All claims expressed in this article are solely those of the authors and do not necessarily represent those of their affiliated organizations, or those of the publisher, the editors and the reviewers. Any product that may be evaluated in this article, or claim that may be made by its manufacturer, is not guaranteed or endorsed by the publisher.

## Footnotes

1
https://img.jgi.doe.gov/

2
https://www.metaboanalyst.ca/home.xhtml
