# Supplementary material for: Long-Term Mootral Application Impacts Methane Production and the Microbial Community in the Rumen Simulation Technique System
Source: Front Microbiol. 2021 Oct 8;12:691502. doi: 10.3389/fmicb.2021.691502 (PMC8531547; doi:10.3389/fmicb.2021.691502)
Supplement: Supplementary file 1 [file Data_Sheet_1.zip › Supplementary Material Tables.pdf]

Supplementary Material

1 Supplementary Tables

Supplementary table 1. Crude nutrients of the substrate.

	**Concentrate[1]**	**Hay[2]**	**Mootral**
	Content of the original substance [%]	Content of the original substance [%]	Content of the original substance [%]
Water[3]	8.7	8.1	7.2
Crude ash[4]	6.7	6.4	3.9
Crude protein[5]	18.5	8.6	22.4
Crude fat[6]	3.2	1.8	0.5
Crude fiber[7]	6.4	29.5	1.9
N-free extractives	56.5	45.6	64.1

[1]Ingredients: rye, wheat gluten feed, rapeseed meal, palm kernels as expeller, beet vinasse, corn gluten feed, calcium carbonate, oat hulls, beet molasses, sodium chloride; vitamins and minerals per kg : 10 000 I.U. Vitamin A, 800 I.E. Vitamin D3, 0.3 mg Se, 0.2 mg Co, 0.1 mg I, 30 mg Zn, 20 mg Mg

[2] second cut, meadow hay

[3] analyzed according to VO (EG) 152 Appendix III, A; 2009

[4] analyzed according to VO (EG) 152 Appendix III, M; 2009

[5] analyzed according to VO (EG) 152 Appendix III, C; 2009

[6] analyzed according to VO (EG) 152 Appendix III, H; 2009

[6] analyzed according to VO (EG) 152 Appendix III, I; 2009

Supplementary Table 2. Internal standard mixture for GC x GC qMS. The used compounds were all at least at 95% purity. The compounds were dissolved in hypergrade LC/MS methanol (Merck KGaA, Darmstadt, Germany).

**Internal Standard**	**Concentration in first extraction solution (80% methanol)**
2-Chlorophenyl acetic acid[1]	20 µM
5-Brom-2,4-dihydroxybenzoic acid[2]	35 µM
p-Chloro-phenylalanine[2]	50 µM
Phenyl-β-D-glucopyranoside[3]	10 µM
Pinitol[1]	10 µM
Sucralose[1]	10 µM
Adonitol[3]	10 µM
Deoxy-Ribose[3]	20 µM
Bromooctadecane[3]	50 µM
13C-Glucose[3]	20 µM
Dibromophenol[4]	20 µM

[1]Acros Organics, VWR, Darmstadt, Germany

[2]Alfa Aesar, Thermo Fisher Scientific, Kandel, Germanys

[3]Sigma-Aldrich, Merck KGaA, Darmstadt, Germany

[4]Supelco, Merck KGaA, Darmstadt, Germany

Supplementary Table 3. Instrumentation, software and measurement parameters for GC x GC qMS analysis

**Instruments and software**	**Name**	**Manufacturer**
Gas chromatograph	GC-2010	Shimadzu Corp, Kyoto, Japan
Mass spectrometer	QP2010 Ultra	Shimadzu Corp, Kyoto, Japan
Auto sampler	AOC-20s	Shimadzu Corp, Kyoto, Japan
PTV Injector	OPTIC-4	GL Sciences, Eindhoven, The Netherlands
Modulator	Zoex ZX1	ZOEX Corp., Houston, USA
GCMS instrument software	GCMS Solution 4.45	Shimadzu Corp, Kyoto, Japan
PTV software	Evolution Workstation 4.1	GL Sciences, Eindhoven, The Netherlands
GC×GC visualization software	GC Image 2.7	GC Image, LLC, Lincoln, Nebraska
**Parameters**	**Setting / value**	
**GC parameters**		
Carrier gas	Helium	
Liner	Deactivated borosilicate glass liner (ID 3.4 mm) with quartz wool (CS-Chromatographie)	
^{1}D column	Rxi-5Sil MS, L = 30 m plus 10 m Integra Guard column, ID = 0.25 mm; film thickness = 0.25 µm (Restek)	
^{2}D column	BPX50, L = 2.6 m, including a “separation segment” of L = 1.1 m, ID = 0.15 mm, film thickness = 0.15 µm (SGE)	
Column connector	SilTite MicroUnion (SGE)	
GC temperature ramp	80.0°C →2.5°C/min→150°C→3°C/min→240°C→17.0°C/min→320°C (hold 7min); total run time 69.7 min	
GC mode	Linear velocity (35)	
Injection mode	Split	
Injection volume	1.0 µL	
Split ratio	1:3	
PTV temperature ramp	40°C→10°C/s→280°C, hold until end of run	
Interface temperature	280°C	
**Modulation parameters**		
Modulator type	Cryogenic, air-based, loop-type	
Modulation period (P_M)	2.9 s	
Cold jet flow	8 l/min	
Hot jet temperature	Programmed stepwise, at least 50°C above oven temperature until 350°C	
Hot jet duration	375 ms	
**MS parameters**		
Ion source temperature	200°C	
Ionization mode	EI (70 eV)	
MS Mode	Scan	
Scan speed	20.000 amu/s	
Scan range	m/z 60-550	
Event time	30 ms	
Data acquisition frequency	33 s^{-1}	

Supplementary Table 4. Significantly different compounds in the metabolome analysis. For the comparisons among ST and LT group the 20 annotated compounds or compound classes with the highest VIP scores were selected and summarized. Green coloring indicates compounds that were represented in both comparisons; blue indicates that the compound was among the 20 highest VIP scores only for LT group and orange only for ST group.

Feature	VIP ST	VIP LT	Compound name/compound class	Similarity Index (with NIST)	Level of metabolite annotation[1]
A0002	1.61	1.27	Propylene glycol	90	2
A0042	1.71	1.97	Hexanoic acid (putative Caproic acid)	88/89	2
A0220	1.72	1.48	2-Hydroxy-3-methylbutyric acid	93	2
A0292	1.63	1.89	2-Methyl-3-hydroxybutyric acid	92	2
A0371	1.66	2.08	Hexanoic acid (putative 2-Hydroxycaproic acid)	91	2
A0399	1.71	1.83	2-Methyl-4-hydroxybutyric acid	91	2
A0533	1.39	2.00	Phenol derivative (putative 4-Isopropylphenol)	82	2
A0673	1.76	1.67	Phenoxyethanol	89	2
A0725	1.71	2.28	Resorcinol	90	2
A0846	1.61	1.51	Indole 1	81	3
A0927_H2	1.60	1.31	Benzenepropanoic acid	77	2
A0948	1.48	2.33	Phenol derivative		3
A0977	1.55	2.35	Indole derivative		3
A1003	1.61	1.79	Tetrose 1	84	3
A1021	1.66	0.98	Tetrose 2	87	3
A1101	1.46	1.96	Hexanedioic acid	73	2
A1188	1.45	1.84	Undecanoic acid	92	2
A1219	1.59	1.70	Tyrosol	94	2
A1409	1.39	1.91	3-(4-Hydroxyphenyl)-1-propanol 1	82	2
A1525	1.54	2.04	Levoglucosan	85	2
A1560	1.48	2.21	Tridecanoic acid 1	91	2
A1728	1.66	1.88	5-Hydroxytryptophan	88	2
A1777	1.70	1.81	Methyl 13-methyltetradecanoate	86	2
A1884	1.74	1.90	Pyridoxine	94	2
A2007	1.59	2.29	Sugar (putative polyol)	80	3
A2200	1.56	2.09	Phytol	80	2
A2297	1.01	1.94	Disaccharide	82	3
A2403	1.44	1.91	Sterol derivative	78	3
A2411	1.71	2.17	beta-Sitosterol	87	2
A2413	1.41	2.00	Stigmastanol	87	2

[1] Identification level according to the Metabolomics Standards Initiative: Identified (1), putatively annotated (2), characterized (3).
