# Supplementary material for: Long-Term Mootral Application Impacts Methane Production and the Microbial Community in the Rumen Simulation Technique System
Source: Front Microbiol. 2021 Oct 8;12:691502. doi: 10.3389/fmicb.2021.691502 (PMC8531547; doi:10.3389/fmicb.2021.691502)
Supplement: Supplementary file 1 [file Data_Sheet_1.zip › Supplementary Material Figures.pdf]

Supplementary Material

1 Supplementary Figures

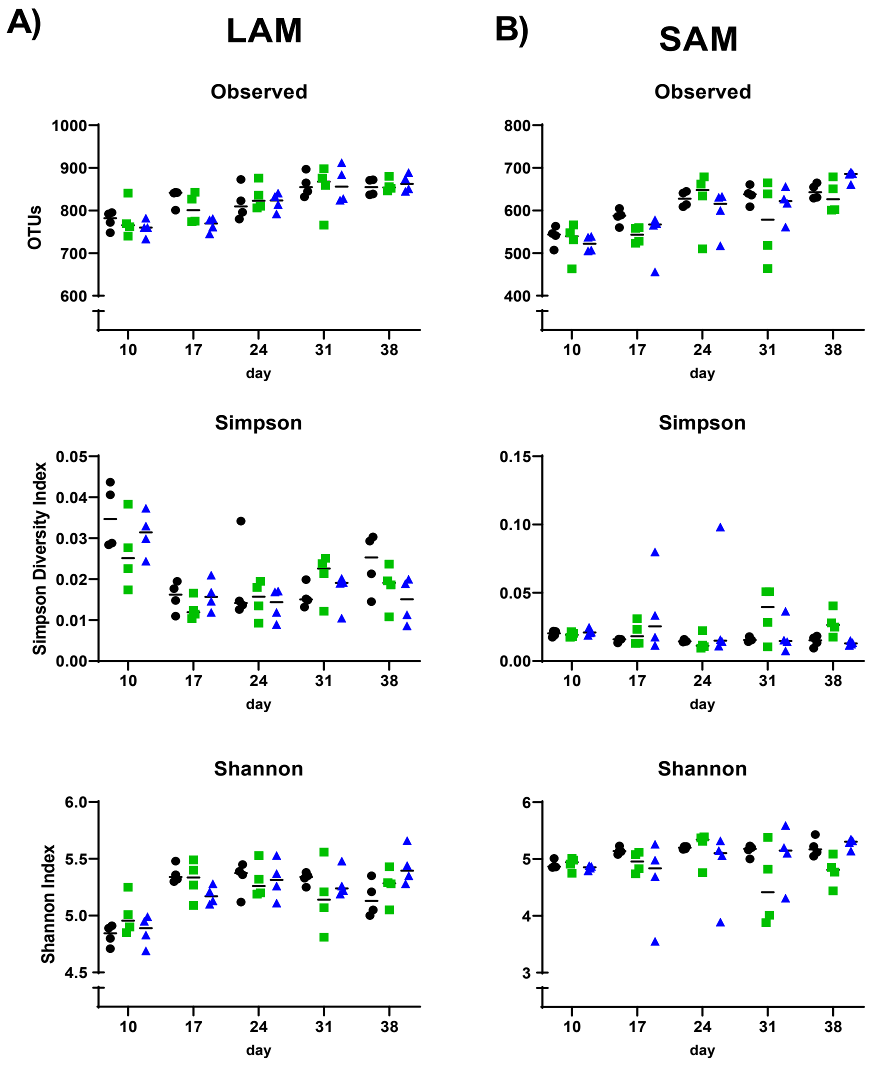

Supplementary Figure 1: Alpha diversity of Bacteria. Number of observed operational taxonomic units (OTUs), Simpson Diversity Index and Shannon Index in LAM (A, left) and SAM (B, right) in the three different treatment groups (n = 4). The fermentation vessels of ST (short-term, blue) and LT (long-term, green) group were supplemented with 1.7 g Mootral each day from day 11 on. The ST group received Mootral until day 27. In CON group (Control, black) no Mootral was added. Lines indicate the median. Two-way repeated-measurements ANOVA followed by Tukey post-test revealed

significant effects of the factor time (LAM all for all indices $P < 0.001$ and SAM for observed OTUs $P < 0.001$).

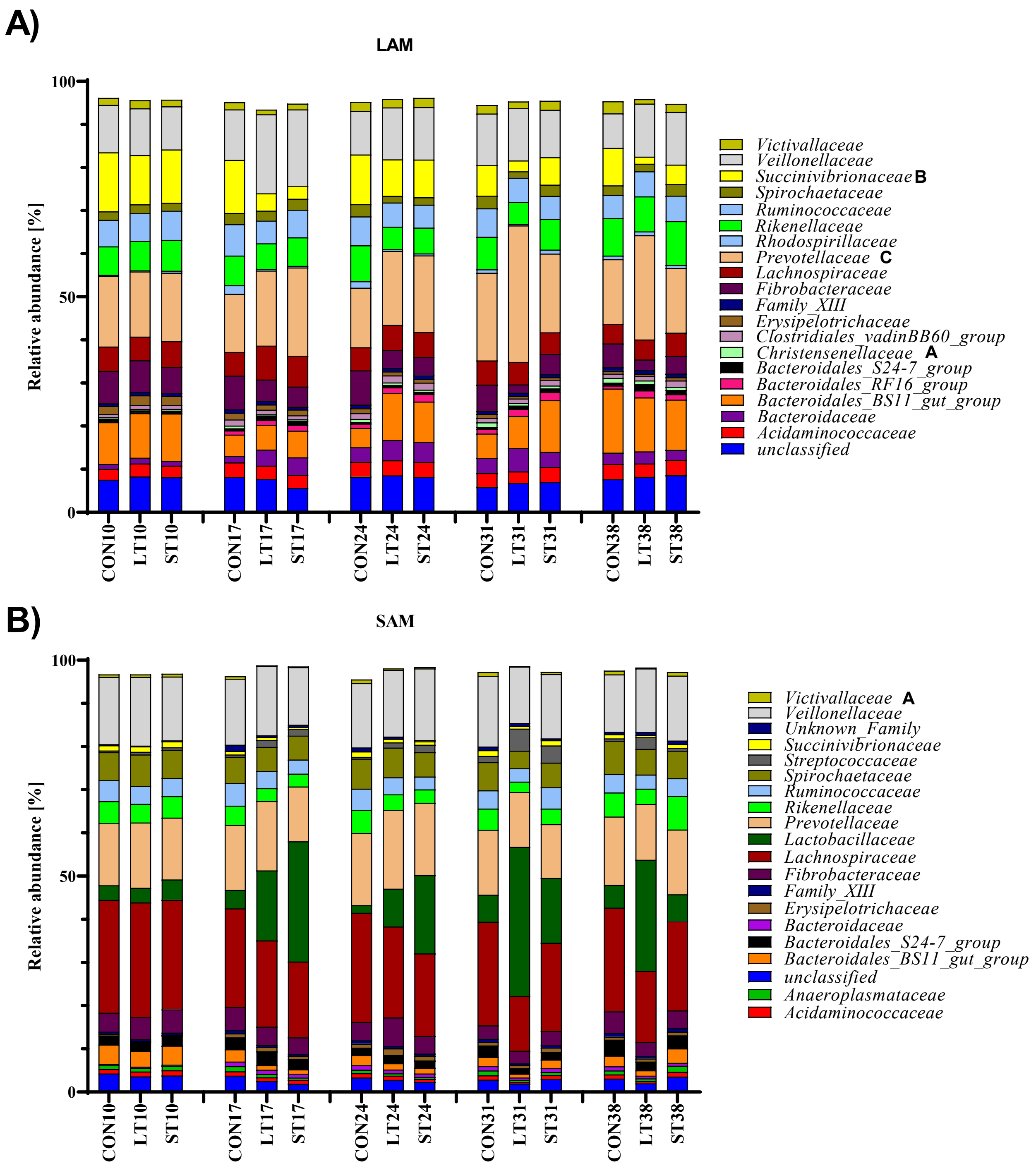

Supplementary Figure 2. Relative abundances of the 20 most abundant bacterial families in LAM (A) and SAM (B) samples for the three treatments at different time-points. ST: short-term group, LT: long-term group, CON: control group. Data are presented as means. Significant differences revealed by multiple t-test are indicated by the following letters: A (CON17 vs. LT17), B (CON17 vs. ST17) and C (CON31 vs LT31 and CON38 vs. LT 38).

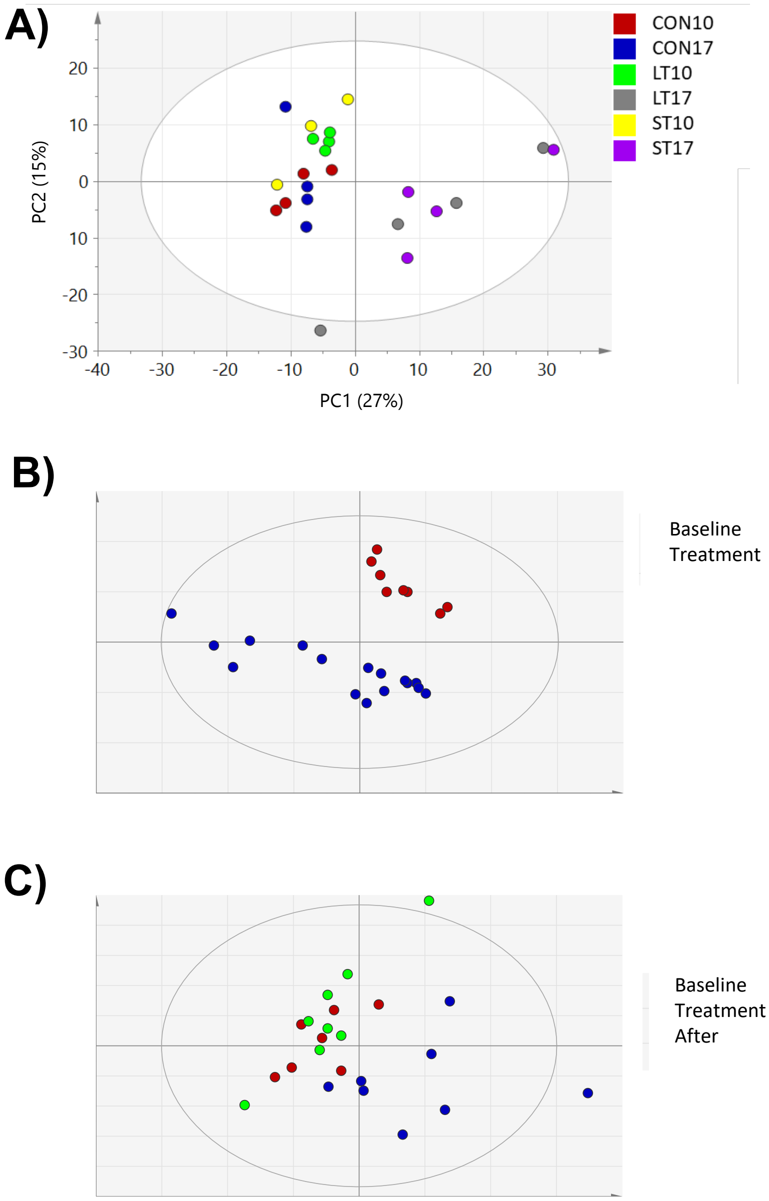

Supplementary Figure 3: Principal Component Analysis (PCA) plot of all three treatment groups on day 10 and 17 (A; CON10 red, CON17 blue; LT10 green; LT17 grey; ST10 yellow; LT17 purple), the LT group (B): CON10 and LT10 (Baseline, red) vs LT17, LT24, LT31 and LT38 (Treatment, blue) and the ST group: CON10 and ST10 (Baseline, red) vs ST17 and LT24 (Treatment, blue) vs ST31 and ST 31 (After treatment, green).

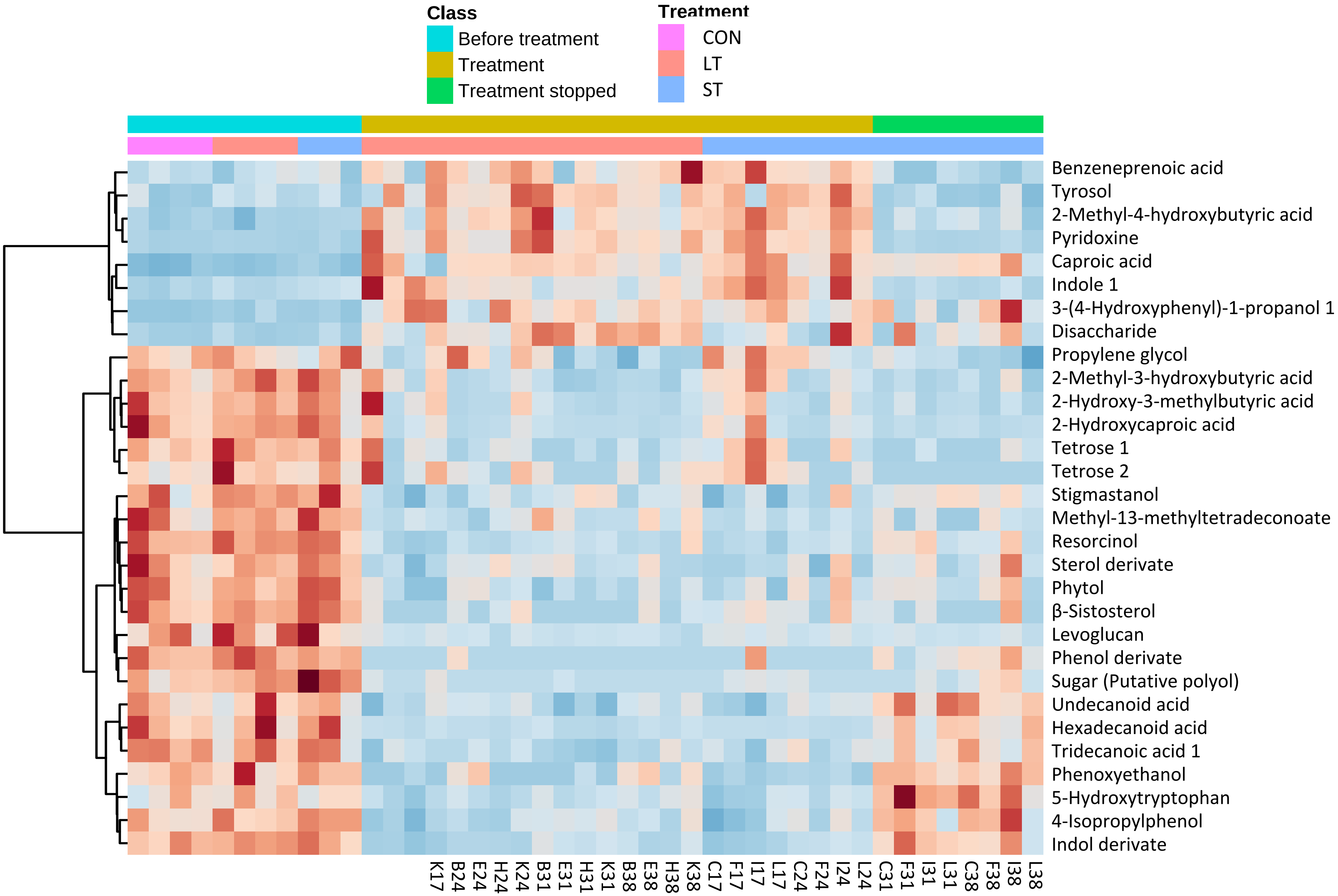

Supplementary Figure 4. Heatmap of the compounds, which exhibited the highest VIP scores for differences among ST and LT time-points and CON10 and could be identified against the NIST database. Sample names, groups and treatments are given for each sample. Displayed data are normalized (median normalization, unit variance scaled) ion intensities; a positive value (red) indicates an enrichment of a compound in a sample, a negative value (blue) indicates a lower concentration.
